# Quantifying the wildfire contribution to PAH concentrations and health effects

**DOI:** 10.1088/1748-9326/ae687f

**Published:** 2026-05-19

**Authors:** Lexia Cicone, Anthony Y H Wong, Noelle E Selin

**Affiliations:** 1 Department of Earth, Atmospheric, and Planetary Sciences, Massachusetts Institute of Technology, Cambridge, MA, United States of America; 2 Center for Sustainability Science and Strategy, Massachusetts Institute of Technology, Cambridge, MA, United States of America; 3 Institute for Data, Systems, and Society, Massachusetts Institute of Technology, Cambridge, MA, United States of America

**Keywords:** polycyclic aromatic hydrocarbons, fires, GEOS-Chem, air quality, toxics, public health

## Abstract

Wildfires are an increasingly important source of toxic air pollutants, including carcinogenic polycyclic aromatic hydrocarbons (PAHs), with implications for human health under a changing climate. Here, we quantify the influence of fire events on PAH concentrations, chemistry, and associated incremental lifetime cancer risk (ILCR) using a global three-dimensional chemical transport model. Fires contribute up to 42% of local PAH concentrations in fire-prone regions, including Australia, sub-Saharan Africa, Siberia, and Canada, and account for approximately 4.6% of global total PAH concentrations. Fire emissions also enhance fine particulate matter (PM_2.5_) levels, promoting partitioning of PAHs into the particle phase. Additionally, fires alter atmospheric oxidant levels, increasing the formation of highly toxic PAH degradation products, which contribute up to 55% of fire-sourced PAH cancer risk. As a result, fire-derived PAH mixtures are 51% more carcinogenic per unit mass than non-fire mixtures. Globally, fires contribute between 4 and 6% of the total all-source PAH ILCR, with some regions exceeding the recommended risk threshold of $1.0 \times 10^{-6}$ due solely to fire-related pollution. Sensitivity analyses indicate that even when fire emissions are dominated by less toxic lower molecular weight PAHs, fire-sourced PAHs are more toxic than anthropogenic-sourced PAHs, highlighting the critical role of atmospheric chemistry in modulating health impacts. These findings demonstrate that wildfires not only elevate PAH concentrations but also increase their per-mass toxicity through chemical transformations. With projections of increasing wildfire frequency and intensity under climate change, our results underscore the need for comprehensive monitoring, emission characterization, and mitigation strategies to address the growing public health risks of fire-related air pollution, particularly in regions frequently affected by wildfires.

## 1. Introduction

Climate change, which is associated with increasing warm and drought conditions, plays an important role in the observed increases in fire-sourced pollutants, like fine (aerodynamic diameter $\unicode{x2A7D}$2.5 *µ*m) particulate matter (PM_2.5_) [1, 2]. From 2000 to 2021 the global average population-weighted fire-sourced PM_2.5_ exposure is estimated to have increased by 65% from 1.85 *µ*g m^−3^ to 3.04 *µ*g m^−3^, with annual premature deaths rising from 156 000 to 241 000 [3]. In regions such as North America, Southeast Asia, and parts of Africa, wildfires have become the single largest contributor to PM_2.5_ health effects [4]. Additionally, wildfire-sourced PM may be more toxic than PM from other sources, potentially causing greater health impacts than previous estimations [5, 6]. The increasing wildfire activity and the associated emissions necessitate the need to better understand the chemistry and composition of wildfire-sourced air pollutants.

One particularly harmful class of pollutants emitted by wildfires are polycyclic aromatic hydrocarbons (PAHs), which are carcinogenic and mutagenic [7]. PAHs are characterized by their multiple fused aromatic ring structure. PAHs consist of hundreds of molecules whose size and structure affect their behavior and toxicity. In the atmosphere, PAHs partition between the gas and particle phase, with low molecular weight (LMW) PAHs preferentially partitioning into the gas phase and high molecular weight (HMW) PAHs preferentially partitioning into the particle phase. For this study, LMW PAHs are defined as those containing two to four aromatic rings, while HMW PAHs contain five to six rings.

PAHs are emitted as a complex mixture from incomplete combustion. Globally, total PAH emissions peaked in 1997 and have since declined due to decreasing anthropogenic emissions [8, 9]. Conversely, global fire emissions have increased, making this sector increasingly important to understand [10]. Wildfires can inject PAHs into higher altitudes, contributing to their long-range transport and global air quality issues [11, 12]. In the atmosphere, PAHs can be oxidized into degradation products, which can be more toxic than their parent compounds [13, 14].

Previous observational studies have shown that fires contribute to elevated levels of LMW PAHs and represent a significant source of PAH air pollution. Ghetu *et al* [15] reported that during the 2018 western US wildfire season, average indoor and outdoor gas-phase concentrations of LMW PAHs were approximately three times higher during wildfires compared to pre-fire and post-fire periods. They also found that both cancer and non-cancer health risks associated with PAHs were three times higher indoors and up to 36 times higher outdoors during these events. Silberstein *et al* [16] analyzed ash from homes affected by the Marshall Fire in Colorado and observed elevated PAH levels, with a greater proportion of LMW PAHs. Building on these studies, Dresser *et al* [17], showed that indoor gas-phase PAHs associated with fire events tend to be dominated by LMW PAHs.

The molecular structure of individual PAHs plays an important role in determining the toxicity of fire related emissions, in particular the distinction between LMW and HMW PAHs. Observations suggest that three- and four-ring PAHs are primarily associated with biomass burning, while five- and six-ring PAHs are linked to gasoline and diesel combustion [16, 18, 19]. This reflects combustion temperature differences, as lower-temperature processes like wildfires generate more LMW PAHs [20]. Although general trends in LMW and HMW PAH production from fires are understood, the exact composition, including the relative amounts of individual PAHs remains uncertain. This variability is important for accurately characterizing emissions and assessing their health impacts. HMW PAHs are potentially more toxic due to their greater hydrophobicity and increased tissue partitioning [13].

Fires also impact the chemistry of PAHs. Fires influence atmospheric oxidant levels, such as ozone (O_3_), nitrogen oxides (NO$ _{{x}}$), nitrate radical (NO_3_), and hydroxy radical (OH). Fires contribute about 4% of global total NO$ _{{x}}$ emissions [21, 22]. Additionally, fires emit non-methane volatile organic compounds (NMVOCs), which along with NO$ _{{x}}$, are precursors to O_3_. Some research suggests fires account for 3.5% of global total tropospheric O_3_ production [23, 24]. Within fire plumes, NO_3_ is locally produced by the reaction of O_3_ and NO_2_. NO_3_ can also be directly taken up by aerosols or react with NMVOCs emitted from fires, causing more widespread loss of NO_3_ [22, 25]. The impact of fires on OH is more uncertain. Peng *et al* [26] found that the emission of nitrous acid from fires could be an important local source of OH. However, Coggon *et al* [27] suggest that carbon monoxide and other NMVOCs emitted by fires are OH sinks on a global scale. These fire-induced changes in atmospheric oxidants can alter PAH chemical loss and degradation product formation. In addition, fires are sources of primary and secondary PM, which can alter the gas-particle partitioning of PAHs [28, 29].

Despite growing recognition of health hazards posed by wildfire emissions, significant gaps remain in our understanding of how fires influence atmospheric chemistry, phase partitioning, and health impacts of PAHs globally. While prior observational studies have documented elevated PAH concentrations during specific fire events, comprehensive global modeling quantifying the influence of fire activity on PAHs across different regions and years is lacking. Here we use a global three-dimensional chemical transport model, GEOS-Chem, to quantify the impact of fires on PAH concentrations and health effects, and evaluate how the chemistry of PAHs produced by fires differs from anthropogenic sources. Based on our model, we calculate the magnitude, composition, and spatial distribution of fire-sourced PAH health risks.

## 2. Method

### 2.1. PAH simulation model description

We use a customized version of GEOS-Chem (version 11.01), as described by Kelly *et al* [30], to simulate the global distribution of the 16 United States Environmental Protection Agency (US EPA) priority PAHs, along with degradation products [30]. The 16 EPA priority PAHs are naphthalene (NAP), acenaphthylene (ACY), acenaphthene (ACE), fluorene (FLO), phenanthrene (PHEN), anthracene (ANT), fluoranthene (FLA), pyrene (PYR), benzo[a]anthracene (BAA), chrysene (CHR), benzo[b]fluoranthene (BBF), benzo[k]fluoranthene (BKF), benzo[a]pyrene (BAP), benzo[g,h,i]perylene (BGHIP), indeno[1,2,3-c,d]pyrene (ICDP), and dibenz[a,h]anthracene (DAHA). These 16 PAHs were designated based on their toxicity, prevalence in the environment, and ability to be measured [14].

We assume all PAHs are emitted in the gas phase, and undergo gas-particle partitioning following a polyparameter linear free energy scheme which considers the effects of PAH molecular properties, temperature, and atmospheric PM concentrations [29]. Particle-phase PAHs are separated into organic carbon (OC) and black carbon (BC) components, which are further separated into hydrophilic and hydrophobic components. PAHs undergo dry and wet deposition within and below clouds [31–33]. Oxidative loss from gas-phase photooxidation (+OH), direct nitration (+NO_3_) and heterogeneous ozonolysis (+O_3_) are considered based on a generalized mechanism of PYR from laboratory studies of PYR and similar PAHs. The model accounts for nitro- and dinitro-PYR formation. Ratios of PYR degradation product to parent compound are used to calculate nitro-PAH (NPAHs) and dinitro-PAH (DNPAHs) concentrations, assuming the same ratio across all PAHs.

We run the model at 2.0 degree latitude ×2.5 degree longitude resolution with 47 vertical levels driven by MERRA-2 meteorology [34]. PAHs are fully interactive with other atmospheric species, which are simulated by a coupled HO$ _{{x}}$-NO$ _{{x}}$-VOC-O_3_ chemical mechanism and a bulk aerosol scheme with aerosol thermodynamics calculated using ISORROPIA(II) [35, 36]. Global anthropogenic emissions of non-PAH species follow the Emissions Database for Global Atmospheric Research (EDGAR v4.3.2) [37] and REanalysis of the TROpospheric chemical composition (RETRO v2) [38], with specific regional emissions replaced by the European Monitoring and Evaluation Programme in Europe (EMEP) [39] and the US EPA National Emission Inventory in the United States (NEI11v1) [40]. Biogenic NMVOC emissions are simulated by the Model of Emissions of Gases and Aerosols from Nature (MEGAN2.1) [41] and biomass burning emissions are from GFED4s [42, 43].

### 2.2. PAH emissions and model runs performed

Monthly PAH emissions are taken from the Global Emission Modeling System (GEMS) inventory [9], which accounts for emissions from 8 sectors: residential, agricultural, industrial processes, industrial combustion, transportation, commercial, fires, and electric generation. For anthropogenic sources, activity data is from the International Energy Agency, World Steel Association, U.S. Geological Survey, Nation Master, and United Nations Statistics Division. Fire activity data is from the Global Fire Emissions Database (GFED4s) [42, 43]. Emission factors for each sector and activity are provided by the inventory based on a compilation of existing literature values [9].

We identify a low and a high fire year to assess the range in fire impacts. From the GFED4s database, we chose 2013 as the low fire year ($4.4 \times 10^{13}$ kg dry matter burned), and 2019 as the high fire year ($5.7 \times 10^{13}$ kg dry matter burned) (figure S1). For 2013 and 2019, we run the model for 12 months from January to December following a 2-month spin-up. Anthropogenic and fire emissions follow the simulation year. For both years, we perform two model runs, (i) a baseline simulation and (ii) a no-wildfire (NoWF) simulation with the fire emissions for all species (including PAHs) turned off. The difference between the baseline and NoWF runs is used to isolate the effects of fire emissions.

Meteorological conditions differ between the two years and influence PAH concentrations both directly, through transport and removal, and indirectly through their control on fire emissions. To investigate the direct effects on PAH transport and removal, we performed an additional full year of GEOS-Chem simulation using 2013 emissions with 2019 meteorology, intended as a sensitivity test to help separate meteorological influences from emission-driven variability. For the indirect impact on PAH emissions, fire activity is largely driven by meteorology and therefore emissions are inherently linked to meteorological conditions. To quantify the differences in meteorology between 2019 and 2013 we compute time-mean fields for each year, take the difference between 2019 and 2013, and then average over land.

**Figure 1.**
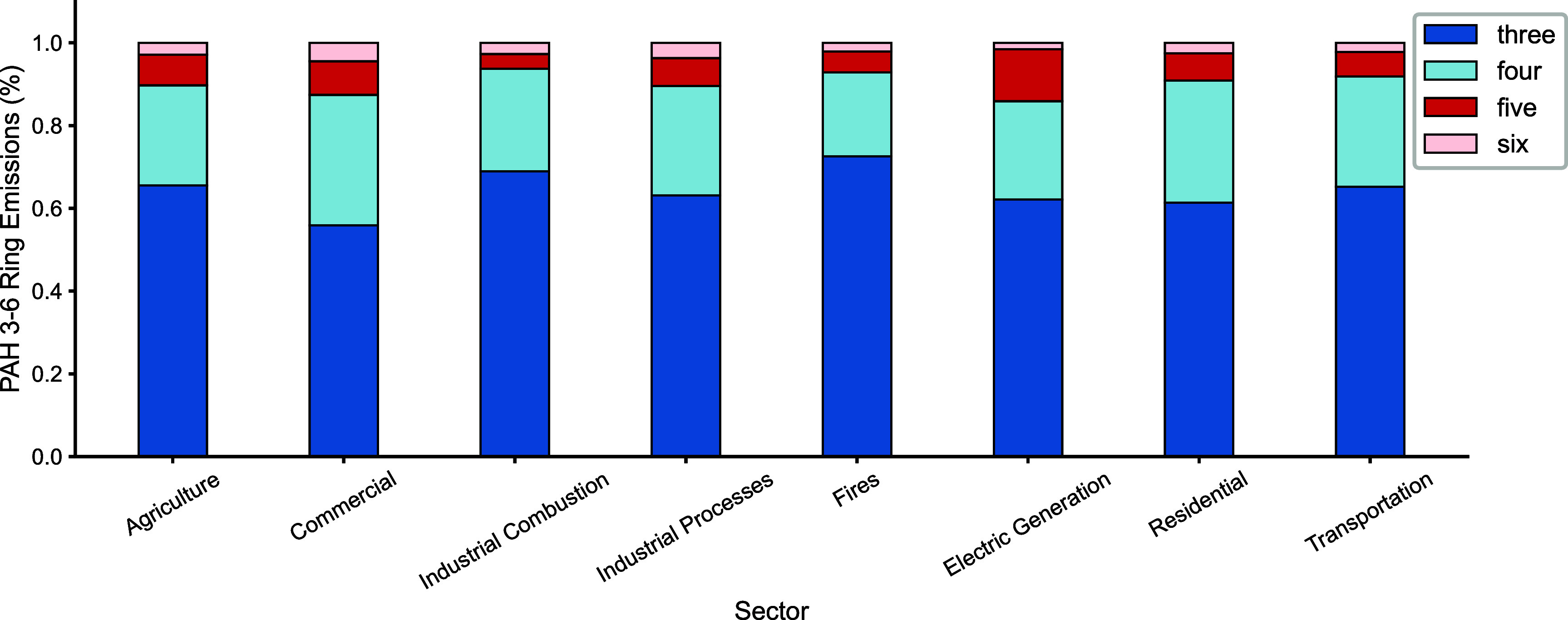
Ring size composition for the eight sectors of the GEMS emission inventory, excluding the two-ring PAH NAP, with dark blue representing three-ring PAHs, light blue representing four-ring PAHs, dark red representing five-ring PAHs, and light red representing six-ring PAHs.

Figure 1 shows the GEMS inventory ring size composition by sector. In GEMS, while some variability in PAH ring-size contributions is present across sectors, the fire sector does not exhibit a distinct composition and lies within the range of other sectors, in contrast to observed sectoral differences in LMW/HMW PAH ratios. We perform an additional run for both 2013 and 2019 to test the sensitivity of our results to assumptions about PAH speciation of fire emissions. To reflect observations that fires preferentially emit LMW PAHs, GEMS fire emissions were modified to include only two-, three-, and four-ring PAHs. NAP emissions were held constant between the original and sensitivity adjusted emissions. For the remaining fire emissions (i.e. the three- and four-ring PAHs), we redistributed the total emissions while preserving the original relative proportions among these species as defined in the GEMS emission inventory. The goal of this sensitivity run is to isolate the effect of changing PAH speciation on concentrations and health impacts, not to represent the true fire emission speciation. First, the annual fire emissions of each individual three- or four-ring PAH species was used to calculate its fractional contribution to the group ($f_{{i}}$):

\begin{align*} f_{i} = \frac{E_{i}}{\sum E}\end{align*}

where $E_{{i}}$ is the original annual emission of species *i*, and Σ*E* is the total annual emission from all three- and four-ring PAHs. These fractions were then applied to the total PAH emission from fires (excluding NAP) to determine the adjusted emissions:

\begin{align*} E^{{\prime}}_{i} = f_{i}\times E_\mathrm{PAH, total}\end{align*}

where ${E}^{{\prime}}_{{i}}$ is the adjusted emission for species *i*, and $E_{\textrm{PAH, total}}$ is the total fire-derived PAH emission excluding NAP. This approach ensures that the total fire PAH emission remains the same as the original inventory while preserving the relative distribution among individual three- and four-ring PAHs.

### 2.3. Calculation of incremental lifetime cancer risk (ILCR)

We use an animal-based method to estimate the ILCR [30]. The ILCR is a unitless value describing the risk that an individual will develop cancer in their lifetime. Although an acceptable risk is subjective, one commonly used acceptable limit is defined by the EPA National Emission Standard for Hazardous Air Pollutants rule as $1.0 \times 10^{-6}$ [44]. The calculation of the ILCR is shown in equation (3). The unit risk factor (UR_A_) describes the increased cancer risk from exposure to a pollutant and is derived from animal-based studies. To capture spatial and temporal differences in PAH composition and attribute health risks to individual PAHs, an essential requirement for this study’s focus on fire-driven changes in PAH mixtures, we follow Kelly *et al* [30] and apply an animal-based method rather than the traditional epidemiological approach that assumes a fixed PAH composition. Health effects are often expressed relative to the reference PAH, benzo[a]pyrene (BAP). For BAP an intermediate value of $1.0 \times 10^{-6}$ per (ng m^−3^) is used for the UR_A_ [45]. In equation (3), the unit risk factor for BAP is multiplied by the atmospheric concentration of the PAH of interest, scaled by its toxic equivalent quotient (TEQ) relative to BAP [30], and then summed over all PAHs. We calculated the ILCR for the 16 EPA priority PAHs as well as 6 NPAHs and DNPAHs with known TEQs.

\begin{align*} \mathrm{ILCR} = \Sigma \mathrm{UR}_\mathrm{A}\times \left[\mathrm{PAH}\right]\times \mathrm{TEQ}.\end{align*}

In addition, we calculate a toxicity per unit mass (TPUM) for all-sourced PAHs (baseline), non-fire-sourced PAHs (NoWF), and fire-sourced PAHs (baseline—NoWF). This is calculated as following

\begin{align*} \mathrm{TPUM} = \frac{\Sigma M_{i}\times \mathrm{TEQ}_{i}}{M_\mathrm{total}}\end{align*}

where $M_{{i}}$ is the surface burden of species *i* (kg), TEQ$ _{{i}}$ is the toxic equivalent quotient for species *i*, and *M*_total_ is the total surface burden of all PAHs (kg).

## 3. Results

### 3.1. Model evaluation

We use observations of all-phase PAHs from continuously monitored networks and individual field campaigns to evaluate our model and assess biases in PAH concentrations. Continuously monitored networks include observations from the US EPA, EMEP, and the National Air Pollution Surveillance program. Individual field campaigns include data from Sweden, Finland, the UK, France, Austria, Italy, Spain, Poland, the USA, Egypt, China, Bangladesh, Cambodia, Japan, Russia, and South Korea [46–55]. For each of the 16 PAHs, we calculate the normalized mean bias (NMB) between modeled and observed concentrations.

**Figure 2.**
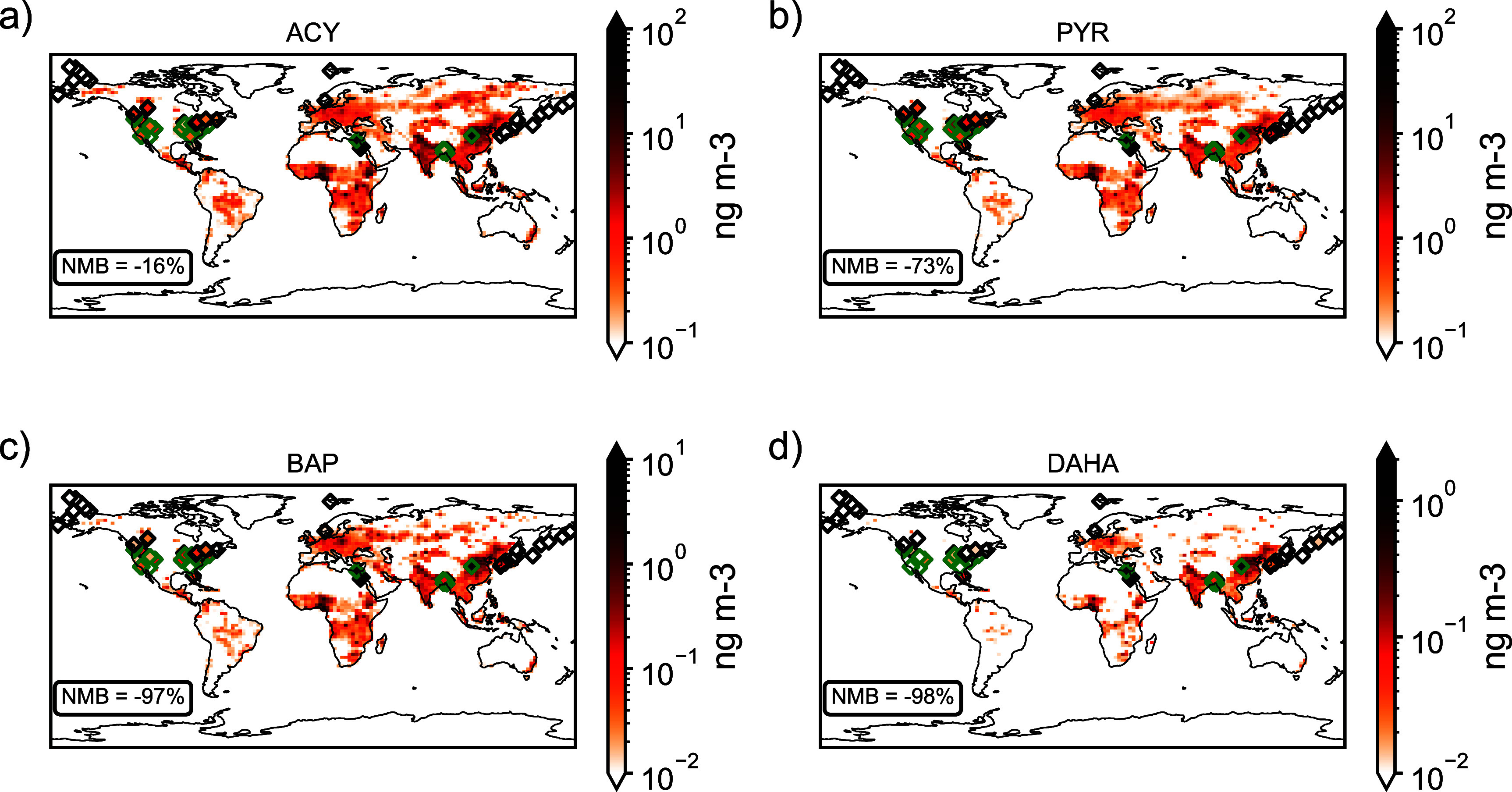
Spatial distributions of 2019 modeled annual mean surface PAH concentrations (ng m^−3^) with observed values plotted in diamonds on top. Green diamonds represent urban observations while black diamonds represent non-urban observations. Panels show 4 of the 16 parent PAHs (a) ACY, (b) PYR, (c) BAP, and (d) DAHA. Note the use of separate colorbar values for each PAH dependent on global burden to improve visualization. The NMB between modeled and observed concentrations is shown on each plot.

Figure 2 shows observations as points plotted over modeled global annual average surface PAH concentrations for 2019. The model is able to reproduce expected PAH hotspots (e.g. China and India) and rural-urban discrepancies in PAH concentration. Limited observations in Africa, South America, and Australia restrict global evaluation. Results are similar for 2013 (figure S4).

In addition to the PAHs shown in figure 2, we calculated the NMB between modeled and observed concentrations for each of the 16 PAHs (table S1). The model underestimates the concentrations of all PAHs, which is a known issue in PAH modeling, and previous studies have suggested that incorporating slower heterogeneous loss processes may improve agreement with observations [56, 57]. Although the inclusion of shielding by coatings of organic aerosol has been suggested as a remedy to slow oxidation, other work finds that this fails to reproduce the observed particulate fraction [57–59]. Underestimation of modeled BC and OC may also contribute to the negative bias by shifting gas-particle partitioning toward the gas phase. Because reactions with OH in the gas phase are the primary sinks controlling PAH lifetimes, this enhances chemical loss and shortens modeled lifetimes, with only a smaller competing effect from reduced deposition [60–63]. This discrepancy remains unresolved. Nonetheless, the PAH model used here produces results that are comparable to previous work [57, 64]. Our results are likely a lower estimate of PAH impacts. The model better captures LMW PAH concentrations with an average NMB of −59% compared to HMW PAH concentrations with an average NMB of −95% in 2019. Because fires emit more LMW PAHs, model bias is likely smaller for this sector. However, because HMW PAHs tend to be more toxic, this discrepancy further contributes to the underestimate of PAH health impacts.

The GEOS-Chem model has been widely used to model fires [65–68]. Despite uncertainty from emission inventory choice, GEOS-Chem is able to capture seasonal mean concentrations, spatial distributions, and vertical profiles of fire-emitted species when compared to surface, aircraft, and satellite observations [69]. In this study, the selected horizontal resolution ($2.0^{\circ} \times 2.5^{\circ}$) is appropriate for our focus on regional and global-scale patterns, as prior work has shown that this resolution reproduces large-scale biomass burning plumes and population-weighted exposure metrics comparably to finer nested simulations, while remaining feasible for multi-year analysis [68, 69]. To investigate the uncertainty in fire-sourced PAHs specifically, we compared total PAH fire emissions from GEMS with those estimated using GFED4s activity data and biomass emission factors from Holder *et al* [70]. For 2019, the GEMS inventory reported total fire emissions of $3.5 \times 10^{7}$ kg, while the Holder-based estimate was an order of magnitude higher at $3.9 \times 10^{8}$ kg. The low fire emissions from GEMS may partially explain the low bias from our model, and imply that the actual contribution of fires to PAH concentrations and health effects could be higher than that represented by the GEMS-based estimates used in this study.

Within this modeling framework, interannual variability in meteorology can affect model output through direct and indirect effects. From the sensitivity test using 2013 emissions with 2019 meteorology, we find that in most regions of the world, the direct impact of meteorology on PAH transport and removal is a minor factor relative to emissions in driving global concentrations for the years tested (figure S2). We also examine the indirect influence of meteorology through its control on fire activity and therefore emissions. Figure S3 shows the difference between 2019 and 2013 for key meteorological variables that can affect fire activity: surface relative humidity, 2 m air temperature, total precipitation, and 10 m wind speed [71]. The results show that 2019 is slightly warmer (+0.44 K) and drier (RH −0.0029), with negligible change in precipitation and a small decrease in wind speed (−0.030 m s^−1^). Spatially, relative humidity decreases are strongest over fire-prone regions such as Australia and parts of South America, while temperature increases more broadly, with the strongest warming signals over Canada and Siberia. These results suggest marginally more fire-prone conditions in 2019, partially explaining why fire emissions are higher in 2019 relative to 2013. While the additional GEOS-Chem simulation using 2013 emissions with 2019 meteorology artificially separates emissions from meteorology, we demonstrate that the direct influence of meteorology on PAH transport and removal is small in present-day climate.

### 3.2. Fire-sourced PAHs

**Figure 3.**
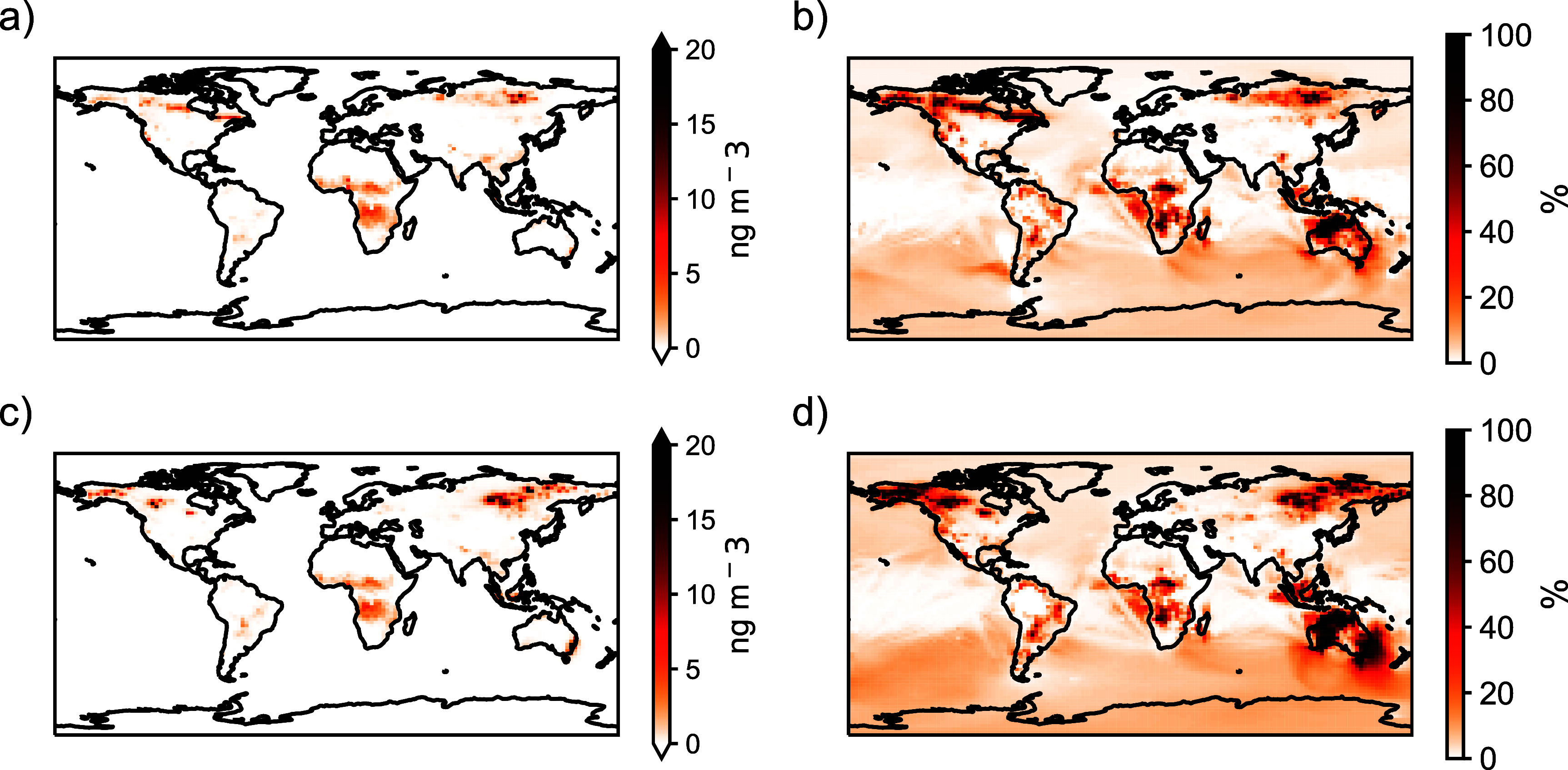
Global contribution of fires to PAH concentrations in 2013 (top) and 2019 (bottom). Panels (a) and (c) show the absolute concentration of PAHs attributed to fires globally while panels (b) and (d) depict the percent contribution of fires to global total PAH concentrations.

In 2019, we find that fires account for 6% of total PAH emissions (4% in 2013). Fires contributed global annual mean surface PAH concentrations of 0.093 (3%) and 0.14 (5%) ng m^−3^ in 2013 and 2019, respectively, with 2019 contributing almost 1.5 times that in 2013. In contrast, total dry matter burned increased by a factor of 1.3 between the two years (figure S1), indicating that changes in PAH concentrations are not simply proportional to total burned mass but are also influenced by other factors like fire location and associated emission factors. Figure 3 shows the spatial distribution of the absolute and relative contribution of fires to total PAH concentrations in 2013 and 2019, respectively. In fire-prone regions like Australia, sub-Saharan Africa, Siberia, and Canada, fire activity contributes an average of 42%, 21%, 17%, and 16% to total PAH concentrations, respectively, and up to 97% locally (figure S6). These fire prone regions are consistent across years (figure S1), illustrating the persistent role of fires there in enhancing local PAH pollution levels.

**Figure 4.**
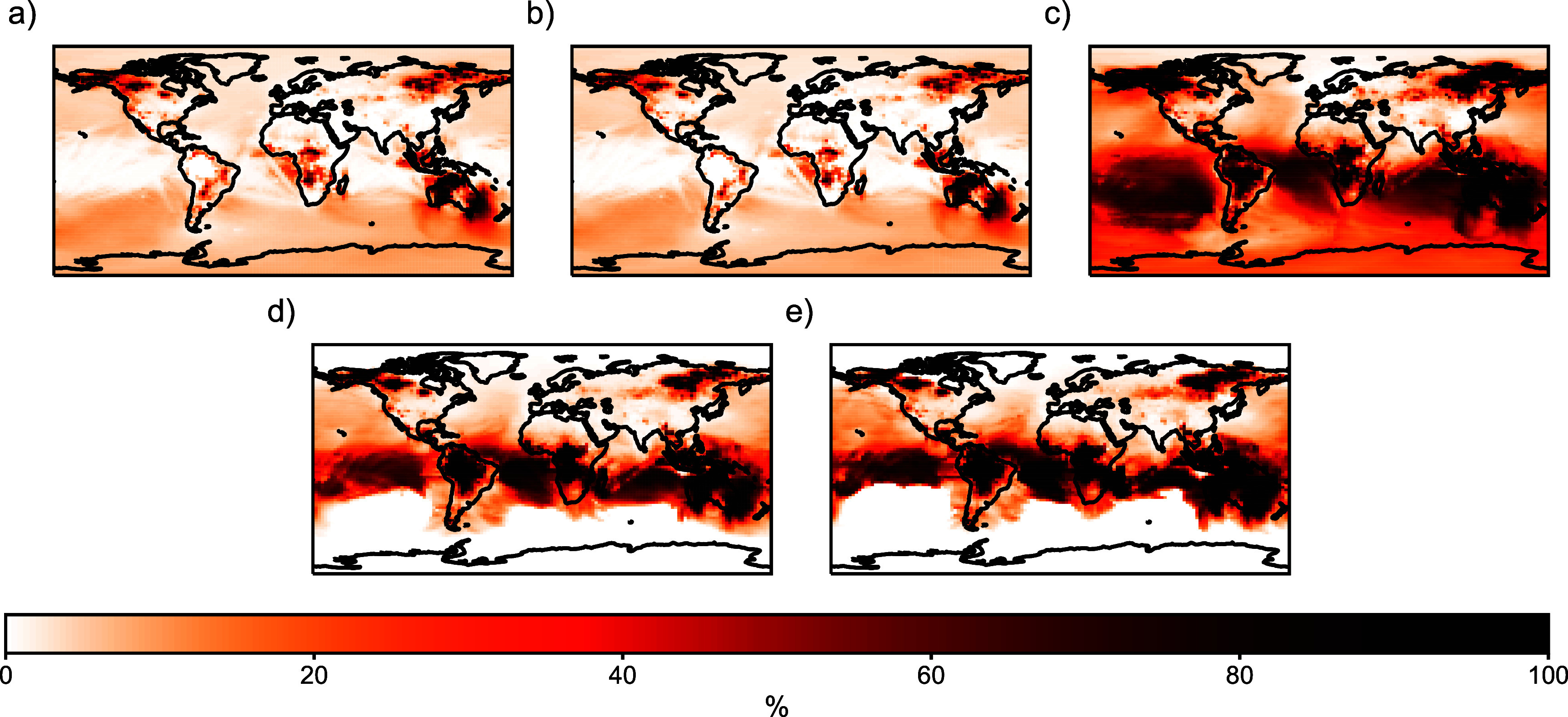
The relative difference between the 2019 baseline and NoWF simulations for (a) all-phase PAHs (b) gas-phase PAHs (c) particle-phase PAHs (d) NPAHs, and (e) DNPAHs. Results for 2013 shown in figure S8.

Fires impact the phase partitioning and degradation process of PAHs. Figure 4 shows the relative difference between the 2019 baseline and NoWF simulations for all-phase PAHs, gas-phase PAHs, particle-phase PAHs, NPAHs, and DNPAHs. Fire emissions induce a larger percent increase in particle-phase PAHs (14%–20%) than gas-phase PAHs (2%–3%). Both NPAHs and DNPAHs have a similar contribution from fires, with the largest signal simulated over the south of the Equator, where OH concentrations are high [72]. A larger fraction (20%) of fire-sourced PAHs is partitioned into the particle phase than all-sourced PAHs (4%). In addition, fire-sourced PAHs are composed of 4% NPAHs and 3% DNPAHs, while all-sourced PAHs are composed of about 1% NPAHs and DNPAHs each. These findings suggest that fire promotes PAH partitioning to the particle phase and the formation of degradation products by locally increasing atmospheric oxidants and PM (figure S11).

**Figure 5.**
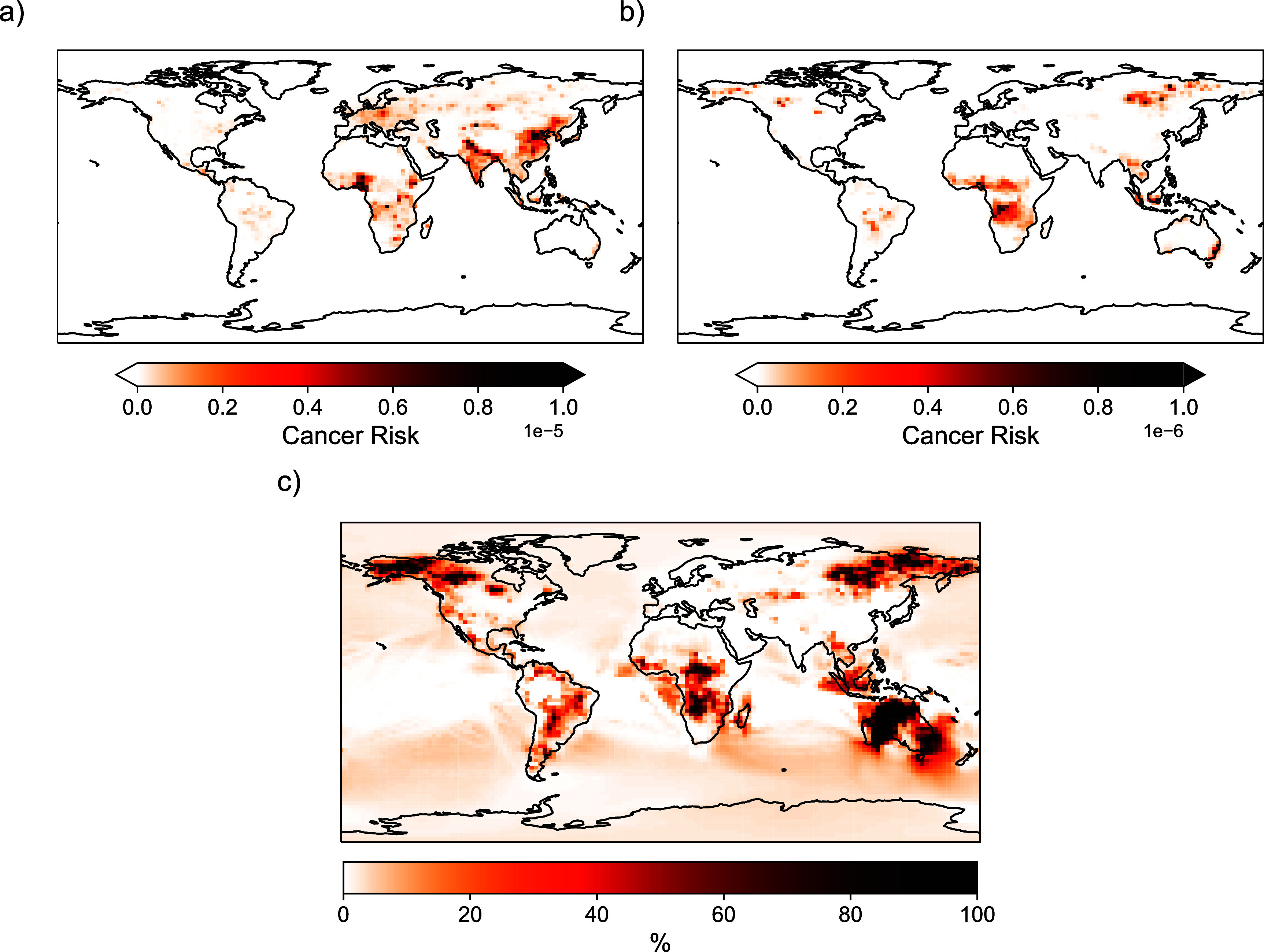
ILCR for 2019 from (a) all-sourced and (b) fire-sourced PAHs. Values to be compared to the the EPA acceptable limit of $1.0 \times 10^{-6}$. Panel (c) shows the percent contribution of fires to global total PAH ILCR. Results for 2013 can be found in figure S9.

Figure 5 shows the ILCR for 2019 from all-sourced and fire-sourced PAHs. For all-sourced PAHs, the cancer risk in certain urban areas, particularly in China and India, exceeds the EPA acceptable limit of $1.0 \times 10^{-6}$. The all-sourced ILCR is approximately an order of magnitude larger than that from fires. Nonetheless, fire-sourced PAHs still contribute to cancer risk, especially in many fire prone regions, with fire-sourced ILCR values exceeding $1.0 \times 10^{-6}$ in Nigeria and Indonesia. Figure 5 panel (c) shows the spatial distribution of percentage contribution of fires to total PAH ILCR, with the highest percentage contribution simulated over Australia, sub-Saharan Africa, Siberia, and Canada. In 2013 and 2019, fires contributed 4.2% and 5.9% of global total PAH ILCR respectively. In fire-prone regions like Australia, sub-Saharan Africa, Siberia, and Canada, fire activity contributes an average of 35%, 34%, 14%, and 16% to total PAH ILCR, respectively, and up to 99% locally (figure S10). For fire-sourced PAHs, 53% of the ILCR is attributed to particle-phase PAHs and 47% to gas-phase PAHs. The 53% contribution from particle-phase PAHs to the ILCR is higher than the 20% contribution of particle-phase PAHs to the total PAH concentration. This disproportionate impact of particle-phase PAHs on health stems from the greater toxicity of HMW PAHs, which preferentially partition into the particle phase. Additionally, the ILCR from degradation products for fire-sourced PAHs is higher than across all sectors. For the fire-sourced ILCR, 22% is from NPAHs and 33% is from DNPAHs. In contrast, the all-sourced ILCR is composed of about 8% NPAHs and 10% DNPAHs.

To explore how chemistry affects the PAH health risk, we compare TPUM values for all-sourced PAHs, non-fire-sourced PAHs, and fire-sourced PAHs. The average values between 2013 and 2019 are $3.5 \times 10^{-2}$, $3.4 \times 10^{-2}$, and $5.2 \times 10^{-2}$ for all-sourced, non-fire-sourced, and fire-sourced PAHs respectively. Fire-sourced PAHs result in higher toxicity due to the co-located local enhancement of short-lived atmospheric oxidants (e.g. OH, NO_3_), which encourages the formation of highly toxic degradation products. This highlights the importance of fire-specific chemistry in increasing PAH toxicity.

### 3.3. Sensitivity test

The sensitivity test replacing five- and six-ring PAHs with three- and four-ring PAHs for the fire sector (figures S12–S14) leads to little difference (0.7%) in modeled total PAH concentrations, with no change in the NMB. The sensitivity runs showed a slightly higher proportion of PAHs in the gas phase for fire-sourced PAHs (82%) compared to the original simulation (80%). This shift can be explained by the greater abundance of LMW PAHs, which more favorably partition to the gas phase. For the sensitivity runs, the overall contribution of fires to total PAH ILCR was lower than the original model runs, contributing 3% in 2013 (compared to 4%) and 5% in 2019 (compared to 6%), which is due to the lower toxicity associated with LMW PAHs. In line with the observed effects on PAH concentrations, the sensitivity runs showed a reduced contribution from the particle phase, which dropped to 40% compared to the original run with 53%. The TPUM values for all-sourced PAHs and fire-sourced PAHs decreased to $3.4 \times 10^{-2}$ and $4.8 \times 10^{-2}$ respectively. However, even when factoring in the higher portion of less toxic LMW PAHs emitted, PAHs from fires are still more toxic.

## 4. Discussion and conclusion

We evaluated how fires affect PAH emissions, concentrations, chemistry, health, and their sensitivity to emission composition. We found that the net effect of increased emissions and changes in atmospheric oxidation is a rise in global annual mean surface PAH concentrations. Fires dominate local PAH concentrations, contributing 97% in parts of Australia and Canada, but 4.6% globally. To understand the range of fire impacts, we analyzed a high (2019) and low (2013) fire year. The magnitude of their effects differed with 2019 contributing almost 1.5 times the concentration in 2013, but the general patterns were consistent across both years.

Fires, as an important source of aerosol, increase the fraction of PAH partitioning to the particle phase, with 20% of PAHs from fires partitioned into the particle phase. Because particle-phase PAHs tend to be more toxic, they are often considered the primary contributors to health risks. However, ignoring gas-phase PAHs can significantly underestimate total exposure, overlooking 47% of the ILCR. Nonetheless, many observational studies exclusively measure particle-phase PAH compounds, and many public health responses to fires emphasize particle-phase exposure. While this study assumes the same TEQ across both phases, emerging evidence suggests that LMW PAHs may contribute to non-cancer health outcomes such as neurotoxicity [73]. To accurately assess the health impacts of PAHs and develop effective mitigation strategies, it is essential to measure, model, and understand their concentrations and behavior across all phases.

Fires increase the contribution of degradation products to total PAH concentrations and health effects. Fires increase NO$ _{{x}}$, NO_3_, and O_3_ concentrations locally, enhancing the formation of more carcinogenic nitro- and dinitro-PAH degradation products. The 6 NPAHs and DNPAHs considered contribute as high as 55% of the fire-sourced ILCR. Therefore, observations and models should incorporate degradation products, not only parent PAHs. Failing to do so risks underestimating health effects by ignoring a substantial portion of PAH toxicity, especially for fires where degradation products play a more significant role in health risks compared to other sectors.

Fire-sourced PAHs pose a significant health risk, with some regions experiencing ILCRs exceeding the recommended threshold of $1.0 \times 10^{-6}$ due to fire-related pollution alone. In particular, Australia, sub-Saharan Africa, and Southeast Asia were found to have the highest PAH health impacts from fires. In addition, fire-sourced PAHs have a 51% higher TPUM compared to non-fire-sourced PAHs. This indicates that the strong local enhancement of PAH oxidants from fires leads to the faster degradation, and therefore higher toxicity for fire-sourced PAHs than anthropogenic PAHs. Our sensitivity test indicates that even if fires have a higher contribution from less carcinogenic three- and four-ring PAHs, fire-sourced PAH mixtures are still more carcinogenic than non-fire-sourced PAH mixtures, showing that wildfire PAH toxicity is governed more by plume chemistry than by primary emission composition. This suggests that fire pollution cannot be treated as chemically equivalent to anthropogenic pollution in exposure assessments.

This study offers an understanding of the impacts of fires on PAH chemistry and health effects on a global scale. However, more observations of all-phase PAHs and their degradation products with global coverage are needed to better constrain the model. This is especially true for the Southern Hemisphere. In addition, future studies on the complex composition of fire emissions from natural and human-made materials are needed to improve exposure estimates. The results of this study, together with the projected increase in fire intensity and frequency due to climate change, and the expansion of the wildland–urban interface, where more people may be exposed to fire-related pollution, and more interactions between fire-sourced PAH oxidants and anthropogenic PAH emissions will take place, highlight the need for comprehensive studies on how fire modulates PAH chemistry, toxicity, and health impacts.

## Data Availability

The data that support the findings of this study are openly available at the following URL/DOI: https://zenodo.org/records/17187658 [74]. Supplementary data 1 available at: https://doi.org/10.1088/1748-9326/ae687f/data1.
